# Development of cognitive remediation interventions for people living with HIV in South Africa: participant experiences of adapted CogSMART and BrainHQ^©^ programs

**DOI:** 10.3389/fneur.2026.1741135

**Published:** 2026-04-29

**Authors:** Nawal Mohamad, Kevin G. F. Thomas, Rhiannon Chernotsky, Reuben N. Robbins, Elizabeth W. Twamley, David E. Vance, Ntombizamantungwa Nyembezi, Zanele Nhlabatsi-Khumalo, Hetta Gouse

**Affiliations:** 1 ACSENT Laboratory, Department of Psychology, University of Cape Town, Rondebosch, South Africa; 2 The Graduate Center, Department of Clinical Psychology, Queens College, City University of New York, New York, NY, United States; 3 Faculty of Humanities, University of Pretoria, Pretoria, South Africa; 4 BRAIN Laboratory, Department of Psychiatry and Mental Health, University of Cape Town, Rondebosch, South Africa; 5 HIV Center for Clinical and Behavioral Studies, Department of Psychiatry, New York State Psychiatric Institute and Columbia University, New York, NY, United States; 6 Department of Psychiatry, University of California, San Diego, La Jolla, CA, United States; 7 Center of Excellence for Stress and Mental Health, VA San Diego Healthcare System, San Diego, CA, United States; 8 School of Nursing, University of Alabama at Birmingham, Birmingham, AL, United States

**Keywords:** cognitive rehabilitation, community-based participatory research, decolonial community psychology, HIV-associated neurocognitive disorder, neuropsychology, South Africa

## Abstract

**Introduction:**

Previous studies suggest that cognitive remediation training (CRT) programs can help address neurocognitive impairment (NCI) and associated functional challenges in multiple populations. However, no such program has been developed for persons living with HIV in low- or middle-income countries (LMICs).

**Methods:**

In this qualitative study, we aimed to describe how South African people living with HIV perceived and experienced the local adaptations of two US-designed CRT programs. We recruited 34 people living with HIV (27 women; age 30–50 years) from community healthcare clinics and a non-governmental organization. All had Xhosa as their home language, resided in low-income communities, and experienced at least mild NCI. Over a 5-week intervention period, participants completed ten 2-h Cognitive Symptom Management and Rehabilitation Therapy (CogSMART-SA) group sessions and 20 self-guided 30-min BrainHQ^©^ sessions. Post-intervention, we hosted nine focus group discussions (3–5 participants each). We applied thematic analyses to the textual contents of those discussions, using decolonial community psychology frameworks (i.e., focusing on community needs and alternative approaches to researching historically marginalized groups, as well as on collaboration and community engagement through exchanges of culturally and socially relevant ideas).

**Results:**

Most participants agreed the CRT programs were enjoyable, improved their mood, and assisted memory performance. They reported that the interventions helped address long-standing challenges (e.g., social exclusion, internalized stigma, and difficulty managing HIV). Many specified that the interventions helped improve self-care (e.g., via physical exercise and stress management techniques, and by ensuring medication adherence) and indicated a sense of increased agency and empowerment in a stigmatizing society. Moreover, they perceived the interactive, group-based aspects of the interventions as a source of social support. Participants described a collaborative learning space that extended into the community, and reported that they maintained friendships post-study.

**Conclusion:**

The findings provide support for using societal and cultural knowledge in creating effective community-based interventions. Using a collaborative approach to co-design the program offered participants opportunities to reflect on identity beyond the intervention and in relation to broader community positionality. This study highlights the value of including participants as co-contributors to the development of CRT programs that are linguistically, culturally, and situationally appropriate for LMIC contexts.

**Clinical trial registration:**

https://clinicaltrials.gov/study/NCT06466642, NCT06466642.

## Introduction

Globally, there are almost 40 million people living with HIV. More than 90% reside in low- or middle-income countries (LMICs), with more than 60% in what UNAIDS reports refer to as the sub-Saharan African region (1, 2).

Even after the introduction and widespread dissemination of effective antiretroviral treatment (ART) for people living with HIV, neurocognitive impairment (NCI; also known as HIV-associated neurocognitive disorder (3)), remains a common comorbidity of HIV. Epidemiological estimates suggest that between 20 and 50% of people living with HIV meet criteria for NCI, with the variation in across study estimates likely being attributable to the application of different diagnostic systems ^NOTEREF _Ref226030999 \f 1^ (4, 5). NCI in HIV is typically associated with deficits in motor speed, information processing speed, attention and concentration, learning, memory, and executive functioning (6–10), as well as with increased risk for early onset of neurodegenerative disorders (e.g., Alzheimer’s disease) (11–17). Severity of impairment ranges from asymptomatic to a severe dementia-type form, with asymptomatic and mild impairment being most common (3, 6).

People living with HIV who present with NCI may also experience behavioral and emotional difficulties (e.g., mood disorders, social withdrawal) and functional challenges (18–20). Challenges include suboptimal ART adherence, impaired instrumental activities of daily living (e.g., driving, managing finances), worse overall quality of life, poor decision-making and greater HIV transmission risk behaviors, increased need for social services, occupational difficulties, and lower employability (21–32).

Although there are no pharmacological treatments specifically targeting NCI in people living with HIV, some pharmacological interventions have been shown to reduce the severity (but, notably, not the prevalence) of HIV-associated NCI. And, despite the global scope of these public health challenges, psychosocial evidence-based treatments are severely limited (7, 33–36). However, a growing body of evidence indicates that cognitive remediation training (CRT) strategies might be helpful (37–42). One such strategy is *compensatory cognitive training* (CCT), a behavioral skills training approach designed to help individuals acquire relevant skills (e.g., using calendars (43)); another is *restorative computer-based cognitive remediation training* (CCRT), an approach based on the idea that attempting increasingly difficult cognitive tasks can strengthen cognitive functioning (44). Most CRT programs were developed and tested in the United States (4, 41, 45). Few, if any, have been developed or adapted for use with people living with HIV in low- or middle-income countries (LMICs) like South Africa, which has the highest incidence of HIV globally (46, 47). Such development and adaptation are essential given (a) differing cultural, linguistic, and socioeconomic characteristics across the global population of people living with HIV, and (b) the fact that LMICs tend to have higher incidences of HIV than the United States.

### The current study

We describe how Xhosa-speaking South African people living with HIV perceived the local adaptations of two US-designed CRT programs, Cognitive Symptom Management and Rehabilitation Therapy (CogSMART (48, 49)) and BrainHQ^©^ (50). We documented participants’ experience of exposure to the programs, explored whether they found them beneficial, and gathered their input on how to improve them. Previous research suggests that including participants as collaborators in co-designing interventions can ensure delivery in a way that resonates with users, thereby increasing their engagement (51–53). Hence, the exit interviews in the current study were designed to elicit participant feedback for future adaptations.

The research program within which this study was embedded sought to adapt those CRT programs for use in Xhosa-speaking South African people living with HIV (49). The overall aim of the program was to deliver interventions to augment current HIV treatment by helping people living with HIV manage their NCI and activities of daily living more successfully that could be validated in an upcoming randomized controlled trial (RCT).

### Theoretical framework

This research is grounded in decolonial community psychology. The colonial project dehumanized those who did not fit the hegemonic mold of white hetero-patriarchal males, stripping these individuals of material resources, subjectivities, and epistemologies necessary for identity construction (54–56). Decolonial work centers on social justice and emphasizes individual beliefs and values. It aims to build agency and self-esteem within a historical context of dispossession and deliberate exclusion from the dominant discourse (57). The framework further views communities as heterogeneous, and recognizes the intersectionality of gender, race, and class when approaching power struggles (58). Hence, in representing communities, there are crucial considerations involving the diversity of knowledge and the value of dialog to expand the boundaries of knowledge.

We used this framework of decolonial community psychology to continue a process of co-creating, through collaborative discourse with community-based informants and participants, context-sensitive and context-specific intervention programs and associated resources (59). Such co-creation and collaboration allow agency to be emphasized; through first-hand contributions to the knowledge-production process, participants and other community members involved in the research can actively resist existing colonial harms.

## Methods

To establish a full context for the data we analyzed, we provide a brief summary of the methods used in the parent study (49) before describing the methods relevant to the current study.

### Parent study

#### Interventions and their adaptation

As described in an previous manuscript, we adapted CogSMART and BrainHQ^©^ (48), for South African Xhosa-speaking people living with HIV. Using the practical principles of community-based participatory research (49), we adapted the contents of CogSMART and selected BrainHQ^©^ tasks for people living with HIV and made additional cultural adaptations relevant to Xhosa-speaking people. Specifically, we involved local healthcare specialists (neuropsychologists, clinical psychologists, occupational therapists) to identify the most appropriate CRT modules/stimulation activities from among the suite of CogSMART and BrainHQ^©^ options. We then asked a focus group of people living with HIV to assist in identifying suitable cultural adaptations for those modules and activities, and worked with a local non-governmental organization to identify a suitable community-based location in which to host the intervention. Finally, we translated the culturally adapted interventions from the original English into Xhosa and trained lay counselors (drawn from the same community as the focus group members) to administer them (49).

CogSMART is a behavioral CRT program developed for people with TBI. The adapted program, called CogSMART-SA, focuses on educating people about neurocognitive sequelae of HIV and on enhancing daily functioning by teaching participants cognitive skills and strategies (e.g., task vigilance, memory techniques, problem solving) and behavioral skills (e.g., calendar use and to-do lists) that can be integrated into daily routines.

CogSMART-SA was structured as an interactive group session led by a lay counselor. Each session explored specific topics of interest (e.g., psychoeducation on HIV-associated neurocognitive disorders, managing sleep and fatigue, reducing stress, and practicing conversational skills). Participants in the intervention arm were given a study bag that included the first CogSMART-SA module in English and Xhosa, a diary, Post-it^©^ notes (‘stickers’), eye masks, earplugs, calendars, and writing materials. Study manuals included problem-solving worksheets, a sleep diary, and relaxation exercises for use in home exercises and for implementing CogSMART-SA cognitive strategies at home.

BrainHQ^©^ (50) is a computer-based CRT program, with instructions delivered individually to participants, that aims to improve cognitive functioning through engagement in repeated, increasingly complex, game-like activities that cover multiple cognitive domains. In this study, we targeted attention and processing speed. The adapted program featured translated instructions and included nine activities (49). Participants were asked to verbalize their feedback (e.g., thoughts, feelings, critiques) in real-time.

#### Participant recruitment and eligibility criteria

We used purposive sampling to recruit people living with HIV from two primary healthcare clinics in Khayelitsha (the largest township in South Africa, with a mix of formal housing and informal settlements).

Individuals eligible for inclusion had to be Xhosa-speaking, aged between 30 and 50 years, HIV-positive, and prescribed antiretroviral therapy (ART). Further eligibility criteria were: (a) ≥7 years of formal education; (b) meeting study-specific criteria for NCI (i.e., ≥1 positive response on the HIV Cognitive Symptom Questionnaire (HCSQ; (60)) and presence of impaired performance on the NeuroScreen cognitive assessment battery (61)); (c) meeting participation criteria on the University of California San Diego Brief Assessment of Capacity to Consent (UBACC; (62)); and (d) consenting to participate in all study activities.

Exclusion criteria were: Significant neuro-medical comorbidities (e.g., schizophrenia, epilepsy, bipolar disorder, multiple sclerosis, intellectual disability, traumatic brain injury (TBI) with a loss of consciousness >30 min); and medical and other conditions that would have impeded full study participation (e.g., undergoing radiation, being legally blind/deaf). A history of cerebral opportunistic infection (e.g., toxoplasmosis) was not an exclusion criterion.

#### Procedure

**Figure 1:**
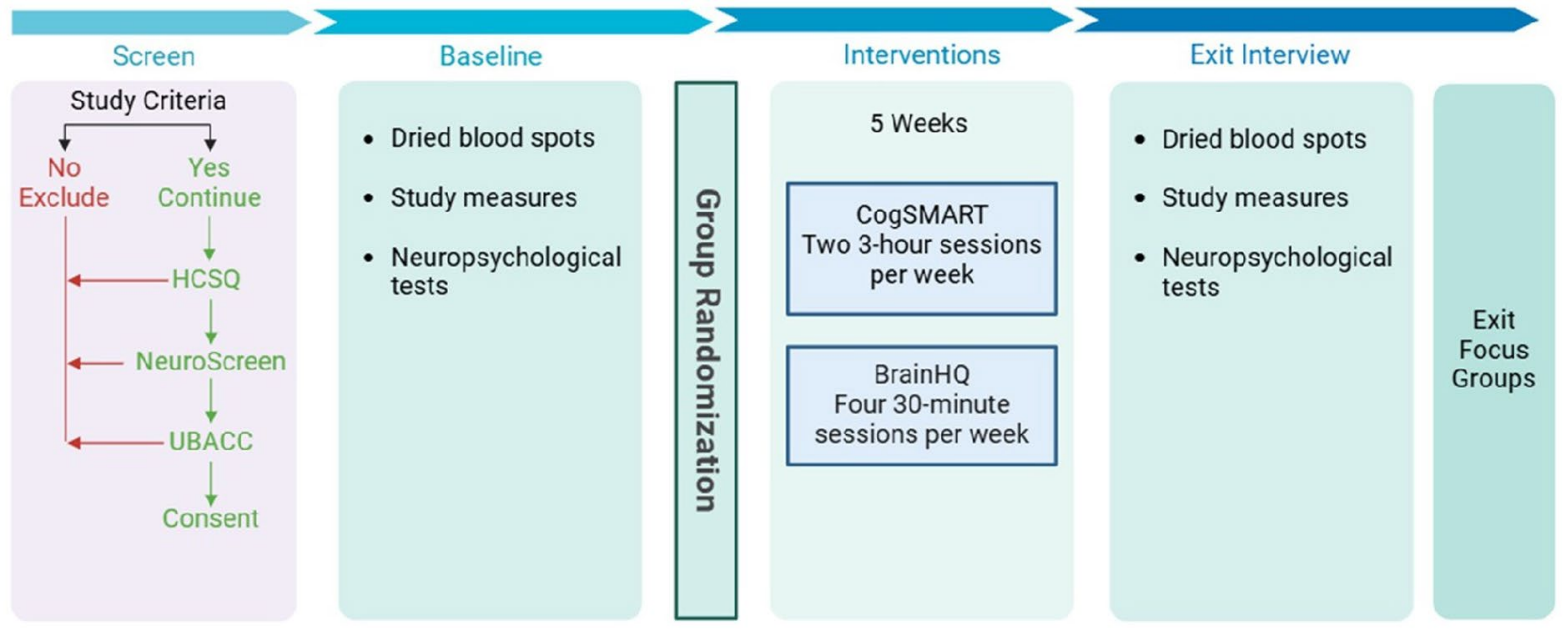
Parent study procedure. The figure presents information about study procedures for the intervention group, from screening for study eligibility to study exit. Regarding the CogSMART-SA sessions, participants were assigned regular homework exercises based on the weekly topic. At the beginning of each session, the exercises were reviewed and challenges that arose were discussed. For participants in the control group, the 5 weeks intervention block was filled with goal-focused meetings (each supervised by a facilitator who was a member of the research team) and self-paced puzzle-based computer games. These meetings and games were designed to have no specific therapeutic benefit; both have been shown to generate minimal attrition rates (86, 87).

The entire study procedure spanned approximately 7 weeks. Figure 1 provides details of the activities across that 7-week block.

### Current study

#### Design

We used a qualitative, exploratory, and community-based participatory research design. The focus group data central to this study were collected between April 2023 and March 2024.

##### Participants

At the end of the parent study’s intervention, a subset of participants from the original sample contributed data to the current study. This subset (*N* = 34) comprised 21 participants who had been assigned to the Intervention group and 13 who had been assigned to the Control group.

##### Measures

###### Interview guide

As part of the developmental process of CogSMART-SA, the interviews sought to identify program strengths and areas for improvement. Hence, using a guide developed in English and translated into Xhosa, the 2-h discussions centered on four topics: (1) motivation for study participation and study engagement; (2) session structure and homework; (3) group dynamics and support; and (4) feedback on the study activities to which the participants had been exposed (for those in the Intervention group, CogSMART-SA/BrainHQ^©^; for those in the Control group, the control activities). Although the guide provided structure, facilitators were free to probe further on issues of interest, allowing for deeper exploration of relevant topics.

##### Procedure

Focus group discussions were held at the university-based research center. A facilitator (bilingual [Xhosa-English] staff member) explained the study purpose and procedures before group discussions proceeded.

Focus group discussions were audio recorded, transcribed, and translated into English. Veracity of the textual data was confirmed using conventional forward and back translation processes (63, 64).

### Ethical considerations

Ethical approval for the entire study (the parent study and the focus group interviews described here) was granted by the University of Cape Town’s Faculty of Health Sciences Human Research Ethics Committee (HREC REF: 045/2022). Before formal enrolment in the parent study, we obtained fully informed written consent from all participants for all procedures related to both the parent study and the current study. Participants were compensated ZAR300 (≈US$16) for participation in the current study’s focus groups.

### Data analysis

#### Descriptive statistics

We extracted participant sociodemographic and medical history data from the relevant parent study measures.

#### Thematic content analysis

We used NVivo software (65) to complete analyses of the transcripts from the focus group sessions.

The theoretical framework of decolonial community psychology guided our analyses of the transcribed textual data. In positioning participants as collaborators in further refining the adapted versions of the interventions, the control activities, and the study materials (e.g., to make them suited for a larger RCT), we sought to identify themes related to their perceptions of which aspects of the programs had worked effectively and which needed improvement. In identifying themes emerging from those transcripts, we were sensitive to participants’ accounts of how living with HIV can increase feelings of powerlessness and how social and political discourse about HIV (as reflected within family and community interactions) can create a complex sense of loss and uncertainty when people living with HIV live in stigmatizing and exclusionary environments (66). Furthermore, we took a critical approach to understanding participants’ perspectives on the social and emotional aspects of HIV (e.g., health promotion, disease management and responsibility, and collective support).

Data were deidentified prior to analysis. Following Braun and Clarke’s (67) six steps of thematic analysis, we (1) read all transcripts to establish familiarity, (2) re-read them to identify codes, (3) searched for themes within the data (i.e., similarities in discussions across groups), (4) reviewed the themes, (5) defined and named the themes, and (6) finalized the analyses. Details of the second and third steps are pertinent; the initial coding led to the creation of a codebook, which served as a reference for the subsequent coding in NVivo. Coding involved an iterative process of comparing and refining codes to identify emerging themes. As coding progressed in NVivo, similar codes were grouped into categories, with new codes emerging from the subsequent transcripts organized into categories, subthemes, and main themes.

Regarding qualitative rigor, author NM completed all initial coding. This included double-coding, where complex passages were assigned multiple codes. To assess discrepancies, authors KGFT, ZKN, and HG assessed and verified the codes. A reminder here that (except when questions related specifically to experiences of CogSMART-SA/BrainHQ^©^) data from Intervention and Control participants were analyzed together, and hence all participant responses contributed to the development of the themes and subthemes. If an emerging theme or subtheme related specifically to elements of the intervention, or elements of the control procedures, we verified that the data underlying that theme came from an Intervention or Control group participant, respectively. The analysis concluded when data saturation was achieved.

## Results and discussion

We conducted nine focus groups. Five groups included participants assigned to the Intervention condition (*n* = 21), with group sizes ranging from four to five. The other four groups included participants assigned to the Control condition (*n* = 13), with group sizes ranging from three to four.

Table 1 presents the sample demographic characteristics. Most participants were female and unemployed; the sample age range was 30–50. The modal duration of HIV diagnosis was 10 years.

**Table 1:** Sample demographic characteristics (*N* = 34).

Variable	*f* (%)	*M*	*SD*
Age		41.67	4.99
Sex			
Female	27 (79.41)		
Male	7 (20.59)		
Employment			
Unemployed	30 (88.24)		
Part-time employed	3 (8.82)		
Full-time employed	1 (2.94)		
Months since HIV diagnosis		168.11	72.76
Months on ART		131.01	59.32

ART, antiretroviral treatment.

Our analyses identified three major themes: reclaiming subjectivities through research participation, collaborating to design future research protocols, and changing orientations toward community. Below, we describe how these themes signify ways participants leveraged the study to improve and understand themselves; how participants used their experience of the programs to make recommendations regarding future interventions; and how participants described their broader experiences living with HIV. Notably, the reports participants made in these regards might be described as decolonial in nature – they were resisting imposed (i.e., colonial) narratives. All quotes below do not use participants’ real names.

### Theme 1: reclaiming subjectivities through research participation

At the personal level, participants reported wanting to improve in several areas related to concerns with living with HIV and associated NCI. Two subthemes emerged: First, participants were motivated to better understand their difficulties with living with HIV. Second, after participating they noticed improvements in their cognitive and behavioral functioning.

#### Motivation to understand the self

Participants highlighted a desire to understand how living with HIV could be understood through a positive and humanizing lens. Most explained that they were attracted to participation for two main reasons, both unrelated to any form of monetary incentive. The first was the possibility of improved memory functioning.

*The recruiter spoke with me at the clinic. She told me that this study is about people who are forgetful, and I told myself that I am going to join it because I am like that.*

Buhle (44, female)

The second was the opportunity to understand mental health within racialized and gendered contexts.

*I was motivated because I wanted to learn more about my mental health. Most of the time, as males or as Black people, we don’t normally take things seriously. And you might get very sick and end up being admitted to mental health institutions. The reason is that we don’t get enough education on our health. I was motivated because I wanted more knowledge about my mental health.*

Sabelo (42, male, Control group)

Sabelo and Buhle’s statements depict a desire to understand their experience of living with HIV. Buhle’s curiosity regarding study participation reflects an awareness of the experience of cognitive difficulties among people living with HIV and an eagerness to explore it within that community. Her statement also suggests excitement at the prospect of a study addressing her primary concerns; she appears to welcome the possibility of exploring them in a group space.

Within our theoretical framework, these participant statements reinforce the notions that the self is relational and that individual wellbeing may be conceptualized discursively within groups so that various community-based influences on identity construction (e.g., social networks, social support, reciprocity, sense of belonging) may be explored (68, 69).

Similarly, within our framework Sabelo’s statement about his motivations may demonstrate consciousness of mainstream (i.e., colonial) discourse on race and gender, which deliberately excludes the colonized body. Certainly, his attraction to the study highlights a desire for agency and also perhaps a desire to resist this exclusionary discourse. Colonial discourse organized society according to race and gender, placing Black men in subordinated and dehumanized positions. Hence, this discourse can contribute to the internalized belief of not needing to “take things seriously” (70). One interpretation of this exchange is that Sabelo is (consciously or unconsciously, purposely or inadvertently) engaging with power in a decolonial society and challenging inequalities that uphold white male domination (71).

#### Reclaiming subjectivities: cultivating the self

For participants in the Intervention group, motivation to continue involvement in the study was strengthened by positive cognitive and behavioral outcomes. When asked about what influenced them to return each week, most mentioned the improvements they noticed as their involvement in the study progressed. In particular, many indicated that the provision of material resources (e.g., computer games, calendars, diaries, earplugs, sleeping masks, pencils) improved their memory, planning, and concentration, which in turn improved organization, medication adherence, financial management, and emotional coping.

*I write on those stickers we had so that we don’t forget what needs to be done.*

Lwazi (39, female)

*I am still using it [diary] to write my and my daughter’s appointment dates[…] When you want to do your homework, you should put those things [earplugs] in because maybe your neighbor is making noise or the children in the house are making noise, and you are unable to concentrate on what you are doing.*

Dumisa (30, female)

These findings, although based purely on self-report data and subjective experiences, are similar to objective data emerging from studies conducted in resource-constrained settings. It appears the provision of material resources, such as food, money, and accessible health services (e.g., social workers, treatment guides, nutritionists) contribute to better medication adherence and improved quality of life (72, 73).

By enrolling in the study and committing to the procedures, participants in the Intervention group gained access to material resources and knowledge that allowed them to manage their health conditions better and to cope more effectively with daily demands. Hence, within our theoretical framework, we allow the speculation that their rebuilt confidence put them in a position to subvert the context of exclusion imposed on people living with HIV and to reclaim positive identities and positions in their community. This finding, then, affirms our decolonial community approach to designing the intervention.

For participants in the Control group, talking to other people living with HIV with similar problems helped (unexpectedly) with medication adherence and stress management.

*We were all under stress, and we didn’t know how to handle it [medication adherence]. During and after the study, we were able to deal with or handle the stresses we had. Now we know what to do and when to take our pills, and we know how to handle our stress.*

Nomzamo (47, female; Control group)

Nomzamo’s comment suggests there may have been cross-contamination of group discussions (i.e., topics addressed by the Intervention groups may have filtered through to the Control groups) or a failure of moderation within the Control groups (i.e., not strict enough monitoring of discussion topics). Future RCTs built on this pilot must incorporate mechanisms to ensure that cross-contamination and moderation failures are avoided.

### Theme 2: collaborating to design future research protocols

Because our intention was to design a fully participatory research program, we sought the collaboration of participants in the future administration of the interventions. Their collaboration in the current study’s procedures built on the collaboration of other people living with HIV, local professionals, and community members in preceding stages of the research (i.e., the initial adaptation and translation of the CRTs). The following suggestions are therefore based on the participants’ experiences in this study.

#### Session structure and resources

Although most participants enjoyed the session structure, they also suggested several possible adjustments: increasing session length, playing ‘games’ after lessons, and ensuring enough computers and space to engage with BrainHQ^©^ without having to wait.

*It would be good if we had enough time so that the facilitator can explain everything. Not that she didn’t explain things to us, but it would be nice to add more time.*

Esihle (50, female)

*Start with the games first, then do the sessions, because the games take longer. We must alternate with the computers because they were not enough.*

Dumisa (30, female)

*Change the venue because the space was small. Secondly, the laptops were not enough so we had to wait for each other to finish; it would be nice to all start [BrainHQ^©^ games] at the same time like we did with the [CogSMART-SA] lessons so that no one would disturb the other.*

Nokuzola (42, female)

#### Scheduling and frequency

Most participants appreciated that the intervention sites were within walking distance of their homes; this allowed them to participate while incorporating their daily exercise. Regarding the optimal meeting times, most agreed that daytime on weekdays would be most suitable – outside of those windows, other responsibilities (e.g., caregiving and housekeeping) would take precedence.

*If I can come Monday to Friday, I wouldn’t mind from 8 am till 5 pm because, over weekends, I am busy with washing and with children. Monday to Friday I won’t have stress.*

Zintle (45, female; Control group)

#### Homework challenges

Some participants reported struggling with completing their homework due to external distractions, lack of privacy, and fear of disclosure.

*I’m staying with my cousins, and we don’t know each other’s status, so I had to hide my books from them. The homework was easy, but I had to do it when they are not at home. I wished I had a place where I could do it. So, it would be better if we were to do the homework after the class is done.*

Buhle (44, female)

However, many also described a sense of enjoyment in doing the homework next to their children, who sometimes assisted them with their tasks. They described a feeling of sadness, that when the study ended they could no longer engage in this shared activity.

*It was easy when my children came from school, so they would be busy with their homework, and I would be busy with mine. I would also sometimes ask them to assist me, and it would be nice with all of us being busy. They would even ask me if I didn't have homework because they were busy with theirs when I wasn't doing it.*

Simthandile (37, female)

#### Other

Regarding the manuals, participants stated that having both Xhosa and English language manuals available was helpful and recommended continued use of both versions. Besides the calendar, diary, Post-it^©^ notes (‘stickers’), and earplugs, the other materials (e.g., eye masks, sleep diary, problem-solving worksheet, and relaxation exercises) were used in moderation.

Some participants expressed interest in finding support for coping with interpersonal struggles not directly related to HIV.

*I wish we can add more sessions and more topics to talk about; for instance, we have challenges of our children using drugs, but I don’t know how this can be done. Our children are on drugs, not just my child. Most of us are in the same boat.*

Mandisa (44, female)

Others indicated they would be open to learning more about the long-term effects of ART.

*We need to use that curiosity to learn and find out if it is the HIV or the ART that is causing people to have memory problems, or is it a common illness [memory impairment] that happens with chronic diseases.*

Nokuzola (42, female)

### Theme 3: changing orientations toward community

At the community level, participants reported wanting to improve in several areas related to interpersonal relationships with other community members. Two subthemes emerged from the data. First, participants were motivated to understand how others lived with HIV. Second, after participating they were motivated to maintain the friendships they had formed with other group members.

#### Motivation to connect with the collective

When asked about reasons for joining the study, participants stated they were curious to meet others who were experiencing similar HIV-related concerns. For instance, they were interested in learning about how others coped with their experiences:

*The reason why I joined the study is because there are few studies that are accommodating depression and people living with HIV. We do have health problems most of the time, so when we meet together and hear from other people, that has changed my way of thinking and of doing things.*

Akhona (47, female)

Participants welcomed the collective nature of the study and appreciated the promise of addressing their feelings of isolation resulting from stigmatizing communities. In particular, the collaborative nature of both the intervention sessions and the focus groups enabled them to conceptualize their identities while sharing their experiences of living with HIV.

*We used to share personal things that happened in our lives, things that we’re not comfortable sharing with our brothers, siblings, family, or neighbors. So, we felt free to talk about our problems during the sessions.*

Sabelo (42, male; Control group)

These findings are similar to those emerging from other studies conducted in low- and middle-income countries. It appears that participants exercise their agency by seeking groups that help them both cope better with living with HIV and resist societal stigma (74–76).

From the perspective of decolonial theory, this motivation to create a collective bond suggests an innate orientation toward community connections and collaborative action. Historically, medical spaces in low- and middle-income communities offered little support (beyond basic care) to marginalized groups. This neglect tacitly acknowledged the privilege of Whiteness and erased the experiences of historically oppressed people. Studies such as this offer an alternative approach, one that is rooted in addressing individual concerns while sharing collective knowledge and thus allowing participants to resist continued isolation and marginalization (77–79).

#### Reclaiming the collective: building community

The interactive nature of the study appeared to reduce social isolation: It provided opportunities for participants to build a community that was maintained even after their study involvement ended.

*I took everyone as family and friends. We would talk about everything, not just sickness, but also about how things were at home. Sometimes, I realized other people’s problems were worse than mine. I wouldn’t say so-and-so was my friend—we were a family. Everyone in the class was a friend to me. We talked about money struggles, boyfriends, girlfriends. The more we shared, the more I resolved some of my problems. Sometimes, I would hear Lwazi express a problem I was also experiencing. I’ve learned a lot from this group. Even when I see Lwazi at the mall, I call her my sister.*

Buhle (44, female)

The support participants received from fellow group members helped boost their self-esteem in an otherwise stigmatizing outside world. Previously, sharing their HIV status tended to produce feelings of alienation:

*We have no-one [families] to support or understand our illnesses, some people have been living with HIV for years, but some families don’t understand or accept them even today.*

Nokuzola (42, female)

Now, they felt a sense of belonging when sharing their problems.

Similarly, some participants described how the knowledge they gained (e.g., from the formal psychoeducational aspects of the intervention, as well as from the more informal discussions they had in the group setting and the acceptance they gained there) helped improve interpersonal relationships:

*The study helped me with my relationship with my brother. I also told them [family members] about the topics we discussed because they would ask me. It helped me, and they realized they were wrong because they didn’t know they were hurting me. They have changed now; even my brother saw that they are not the same people they used to be.*

Zintle (45, female; Control group)

Zintle reflected on the confidence she gained to challenge the negative stereotypes and the beliefs about HIV held by her family. By doing so, she managed to demonstrate the harm those stereotypes were perpetuating and improved her family relationships.

In summary, participants’ commitment to each other allowed them to benefit both individually and collectively. At the individual level, they harnessed their self-confidence and experienced reduced feelings of alienation. At the collective level, they found that in their relations with one another they could subvert the social fabric that commonly marginalizes and isolates people living with HIV.

Hence, engaging in discourse that resists social exclusion harnessed feelings of empowerment in various ways. These findings reflect those from other studies reporting that when stigmatized groups feel dignified and included their quality of life improves (80, 81). Resisting stigmatization and social exclusion means fostering inclusive communities geared toward addressing broader systemic challenges, such as social determinants of health and access to healthcare (82–84).

### Reflexivity

Throughout the research process, study staff engaged in critical reflexivity and considered how our positionality may have impacted the results (85). After the focus groups were completed, the facilitators and authors reflected on their experience adapting the program to the South African context. Here is one such reflection:

As a white, middle-class neuropsychologist working in a supervisory role, I was conscious of the distance between my own background and the lived experiences of the participants. This made it all the more important to approach the work with humility and openness. Watching the facilitators lead sessions with warmth, cultural sensitivity, and skill was a powerful reminder that effective intervention goes far beyond content — it’s about connection, trust, and relevance. It was clear that the group format and local grounding made the content more than just cognitive training — it became something participants could relate to and take with them. This experience deepened my understanding of what it means to support community-based research that is responsive to context and grounded in collaboration.

Two facilitators delivered the program to participants. One explored feelings of helplessness and support from her peer facilitators. She navigated feelings of uncertainty and appreciated the knowledge she gained from the process:

I have gained a lot. When I got home, I will think back on what happened during sessions, then I will phone RC and sleep cause it’s too much. Some cases are unbearable, but of course, the supervision helps a lot. I remember the other day, there was a lady who shared her touching story, and she broke down and cried a lot and loud to a place where I didn’t know what to do. I then sent a message to [the co-facilitator] to come in for support, and everyone in the premises was wondering what was happening, but we managed to calm her down.

As a young woman from a middle-income background and not living with HIV, author NM’s experience analyzing the data included feelings of powerlessness and sadness within the context of an appreciation of her institutional privileges. Although her background may have limited her understanding of the participants’ stories and experiences, the collaborative nature of the study helped bridge this gap. NM was also encouraged by participants’ active resistance to harmful ideas about their identities, their use of the intervention to improve their well-being, and their exploration of new ways of living in their communities.

### Limitations

Three primary limitations of the study’s design and methodology constrain the conclusions we can draw and the implications of our findings. First, the nature of our sample (predominantly female, predominantly single-language Xhosa speakers) and the fact that participants were all recruited from an urban township setting might limit the generalizability of the intervention across contexts. Second, the participatory design of the focus groups may have influenced a social desirability bias and prevented the discussion of more stigmatizing topics. Third, future implementations are systemically restricted by constrained resources, barriers to healthcare access, and a lack of evidence-based guidelines for identifying people living with HIV who also present with NCI.

## Conclusion

Our findings, based on co-designed adaptation of US-developed CRTs and the current study’s focus group data, offer qualitative evidence for the value of using local knowledge (individual, societal, and cultural) to improve community-based cognitive rehabilitation training programs. Specifically, this study highlights the value of including participants as co-contributors to the development of CRT programs that are linguistically, culturally, and situationally appropriate for LMIC contexts.

Using a collaborative approach to first adapt and then refine the programs enabled participants to reflect on their identities beyond the intervention and in relation to, for instance, their families and the broader communities in which they were living. This reflection was beneficial, especially as it was accompanied by knowledge, practical skills, and material resources to help them navigate the experience of living with HIV. Of equal benefit and importance to the research team were the insights and information participants provided regarding program modifications that might improve engagement in future implementations.

For future implementations, CRT program efficacy must be determined via randomized controlled trials (RCT), with intervention content and study methodology both tailored to the local setting. Acceptability and usability among the people using the program must be continually assessed and considered, as must the above-mentioned contextual challenges in adapting and implementing the interventions. Strict adherence to guidelines regarding which topics should not be addressed in the Control group sessions are necessary to prevent a treatment effect in that group (e.g., discussions around ART adherence should be redirected).

In closing, we propose that a commitment to the principles of decolonial community (neuro)psychology can help people living with HIV combat stigma and exclusion and integrate their individual and collective desires to reclaim power and status in their communities. Furthermore, such a commitment can help researchers use CRT programs and similar interventions as a springboard for addressing the multitude of social justice issues that are related to, but extend far beyond, HIV.

## Data Availability

The datasets presented in this study can be found in online repositories. The names of the repository/repositories and accession number(s) can be found at: http://doi.org/10.25375/uct.30203428.
